# Recombinant Human Trefoil Factor 3 Ameliorates Bowel Injury: Its Anti-Inflammatory Effect on Experimental Necrotizing Enterocolitis

**DOI:** 10.1155/2014/634135

**Published:** 2014-02-12

**Authors:** Lei Shi, Pang-Hu Zhou, Juan-Li Xi, Hong-Gang Yu, Bing-Hong Zhang

**Affiliations:** ^1^Department of Oncology, Renmin Hospital of Wuhan University, Ziyang Road 99, Wuhan 430060, China; ^2^Department of Orthopedics, Renmin Hospital of Wuhan University, Ziyang Road 99, Wuhan 430060, China; ^3^Department of Medicine, Wuhan Third Hospital, Wuhan 430060, China; ^4^Department of Gastroenterology, Renmin Hospital of Wuhan University, Ziyang Road 99, Wuhan 430060, China; ^5^Department of Pediatrics, Renmin Hospital of Wuhan University, Ziyang Road 99, Wuhan 430060, China

## Abstract

*Aim*. Recombinant human trefoil factor 3 (intestinal trefoil factor) has been suggested to be partially protective against necrotizing enterocolitis (NEC), but the mechanisms of this protection have not been defined. We investigated whether the protective effects of rhTFF3 are the result of an anti-inflammatory response. *Methods*. The rats were killed on day 4, the distal ileum was harvested for morphological studies and immunohistochemistry for NF-**κ**B (p65), and the amounts of IL-1**β**, IL-6, and IL-10 in the intestinal tissue were measured using commercial ELISA assay kits. *Results*. In the neonatal NEC, IL-1**β**, IL-6, and IL-10 were significantly higher than in normal group. In normal group, IL-1**β** and IL-6 were significantly decreased, and the amount of IL-10 was markedly increased compared with NEC group. In the NEC model, immunohistochemical staining for NF-**κ**B (p65) was demonstrated to be of a strong brown color and was distributed in the intestinal epithelium. Treatment with rhTFF3 significantly decreased the immunoreactivity of NF-**κ**B (p65) in the NEC model. *Conclusions*. Intestinal inflammation was ameliorated after rhTFF3 was injected. rhTFF3 may protect against the intestinal injury of the neonatal rat NEC model by suppression of the inflammatory response.

## 1. Introduction

For the past several years, due to the increasing survival rates of premature infants, the incidence and lethality of necrotizing enterocolitis (NEC) in neonates have increased. NEC remains a leading cause of morbidity and mortality in neonatal intensive care units with a reported incidence of 10.1% among very low birth weight infants (1500 g) [1] and a mortality of 15–30% [2]. A disease with a serious prognosis, advanced cases of NEC may cause multisystem organ failure [3]. Studies in recent years indicate that its pathogenesis is associated with premature delivery, intestinal ischemia-hypoxia damage, immature intestinal tract function, and the organism's resistance, nutrition, and bacterial infection, among other factors [4]. These risk factors are responsible for the production of inflammatory mediators and for activation of the local inflammation cascade; a series of changes occurs at the cellular level, and an overbalanced interaction between the intestinal necrosis proinflammatory cytokines and anti-inflammatory cytokines is triggered. The inflammatory cascade is started, leading to colon tissue immunologic injury, and a series of clinical symptoms appear. Proinflammatory cytokines such as IL-1, IL-6, IL-8, and TNF-*α* and anti-inflammatory cytokines such as IL-4, IL-10, and IL-1ra are closely related to NEC, and they can directly or indirectly impact target cells simultaneously or one after another to form a cytokine network. Unbalanced network control is likely the juncture of the diverse intestinal mucosa pathological changes.

Nuclear factor kappa B (NF-*κ*B) is a transcription factor family member, and it regulates the expression of many inflammatory genes including proinflammatory cytokines, chemokines, and leukocyte adhesion molecules [5, 6]. NF-*κ*B can be activated by various types of stimuli, including tumor necrosis factor TNF*α*, IL-1 inducing inflammatory reaction, T cell and B cell mitogen, and bacteria [7].

TFF3 (trefoil factor 3) is a member of the trefoil factor family and possesses a fairly strong cell-protective function; it is capable of diminishing multiple injury factor-mediated intestinal mucosa damage, and it possesses a cell-proliferative and migrative ability and also has distinct physiologic functions, namely, binding with mucous glycoprotein and stabilizing the intestinal slime layer. TFF3 plays an important roles in enteral self-protection and repair following injury [8].

NF-*κ*B is aberrantly upregulated in many chronic inflammatory diseases, including inflammatory bowel disease, and the inhibition of NF-*κ*B activation has been shown to decrease bowel injury [9]. We previously demonstrated that rhTFF3 has a protective effect on intestinal injury in NEC in an experimental model [10], but the mechanisms of this protection are not yet defined.

Neonatal Wistar rat models were used in this study. A positive control was established by giving rhTFF3 to detect NF-*κ*B expression in enteral histopathological and immunohistological examinations, and the amounts of anti-inflammatory cytokines (IL-10) and proinflammatory cytokines (IL-6, IL-1*β*) following NEC were measured to determine whether the suppressed inflammatory response by rhTFF3 has a protective effect in the development of NEC.

## 2. Materials and Methods

### 2.1. Animals and the Necrotizing Enterocolitis Model

The study was approved by the animal care and use committee of Wuhan University. Fifty 1-day-old Wistar rat pups (5–10 g) whose mothers were maintained under standard conditions were recruited for the present experiment. All the experimental protocols were performed according to the guidelines for the ethical treatment of experimental animals. Local institutional approval was obtained before initiation of the study. As Caplan et al. [11] reported, the NEC model of neonatal rats was established as follows: hypoxia was induced by stressing each rat pup with 100% N_2_ (maintaining (99.96 ± 0.2)%) for 60 s, followed by exposure to the cold (4°C) for 10 min two times daily. The newborn rats were hand-fed via an oral feeding tube with 0.15 mL of formula every 4 hours.

### 2.2. Experimental Design

Fifty rat pups were randomly divided into five groups. In group A (*n* = 10), the rats were the nonhypoxic untreated control group. Group B rats (*n* = 10), according to Zhang et al.'s previous work [10], were injected subcutaneously with rhTFF3 (0.2 mg per rat) without asphyxia/cold stress. Groups C, D, and E (*n* = 10, resp.) rats were subjected to asphyxia/cold stress. During the next 3 days, group D rats were injected with 0.2 mL of 0.9% sodium chloride and group E rats were injected subcutaneously with rhTFF3 (0.2 mg per rat). On the fourth day, all the rats were killed by decapitation. The intestinal tissues located at the boundary of the ileum and the cecum were bathed carefully in a 10% formalin solution for immediate fixation and processed routinely and embedded in paraffin for histological examinations. The administration of asphyxia/cold stress was performed two times daily (8 AM and 4 PM, resp.). To examine the therapeutic effect, the rats were given rhTFF3 by subcutaneous injection 60 minutes after the asphyxia/cold stress finished (4 PM).

### 2.3. Preparation of rhTFF3

rhTFF3 was provided by the Institute of Gene and Protein Research, Beijing University (Beijing, China). As Wang et al. [12] reported, the human TFF3 DNA fragment was amplified by polymerase chain reaction (PCR) from human fetal placenta cDNA. The gene was cloned into the *Pichia pastoris *expression vector pPIC9K containing the *AOX*1 promoter and an *α*-factor leader sequence. Multicopy insertion transformants were screened on G418 plates. After induction by 2% methanol for 48 hours, the expression of dimeric hTFF3 came up to 45% of total proteins in medium, as identified by SDS-PAGE and a western blot assay. The recombinant protein was further purified by S-Sepharose, Q-Sepharose ion-exchange chromatography, and Sephacryl S-100 gel filtration chromatography to the 95% purity, as shown by densitometric scanning. The N-terminus and molecular weight of the rhTFF3 were in good agreement with the native hTFF3.

### 2.4. Histological Study

A representative 1-2 cm long specimen was taken for histological study from the ileum and the cecum. After formalin fixation and paraffin embedding, the processed and sectioned (4 *μ*m thick) tissues were stained by hematoxylin and eosin (HE). A pathologist from the Renmin Hospital of Wuhan University was blinded to the experimental groups and graded the morphological changes in the intestinal epithelium. As Nadler et al. [13] reported, the criteria for each histological grade (0–4) were as follows: normal (0), no damage; mild (1+), slight submucosal and/or lamina propria separation; moderate (2+), moderate, moderate separation of submucosa, and/or lamina propria and/or edema in submucosal and muscular layers; severe (3+), severe separation of submucosa and/or lamina propria and/or severe edema in submucosal and muscular layers and regional villus sloughing; necrosis (4+), loss of villi and necrosis.

### 2.5. Measurement of IL-1*β*, IL-6, and IL-10

In the present study, the cytokine analysis was performed in duplicate using commercial ELISA assay kits (Boguang Biotech, Shanghai, China) for rats IL-1*β*, IL-6, and IL-10 according to the manufacturers' instructions. The results are expressed in pg/mL.

### 2.6. Immunohistochemistry

Formalin-fixed, paraffin-embedded tissue blocks were cut 4 *μ*m thick and mounted on glass slides. After mounting, they were kept in an oven at 70°C for 2 h. The sections were deparaffinized in xylene and rehydrated. The endogenous peroxidase activity was blocked with 3% hydrogen peroxide for 10 min. Antigen retrieval was performed by microwave. An overnight incubation was performed with the polyclonal antibody for NF-*κ*B (Santa Cruz Biotech, Santa Cruz, CA) in 1% phosphate-buffered saline-bovine serum albumin (1% PBS-BSA) at a dilution of 1 : 80. The sections were washed three times with PBS for 2 min each and incubated with biotin-labeled anti-rabbit IgG for 1 h at room temperature. After three washes with PBS for 2 min each, the sections were stained by a streptavidin-peroxidase detection system. Incubation with PBS instead of the primary antibody served as a negative control.

### 2.7. Statistical Analysis

The data were expressed as the mean values ± standard deviation as described in the individual figure legends. The data were analyzed for statistically significant differences with one-way ANOVA. For comparisons of the pathological scores, Ridit analysis was used. A *P* value of <0.05 was considered statistically significant.

## 3. Results 

### 3.1. rhTFF3 Ameliorates Histological Injury in Necrotizing Enterocolitis

**Figure 1:**
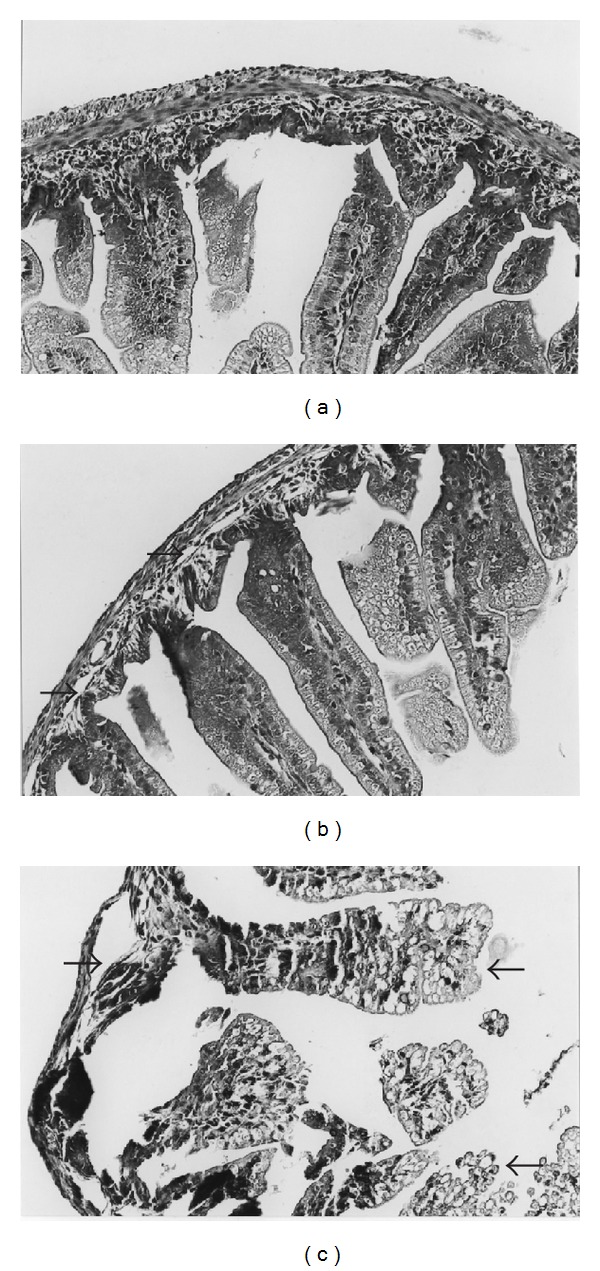
Small intestinal histology in the neonatal rats treated as described in Section 2 (HE staining). (a) Control animal showing the normal intestinal villus architecture (score 0). (b) NEC animal treated with rhTFF3 by subcutaneous injection showing a slight histological injury (score 2): moderate separation of submucosa (arrow). (c) NEC animal with complete villous necrosis (score 4): severe separation of submucosa and loss of villi with necrosis (arrow). (HE, magnification ×200.)

Histopathologically, the normal control rats had no gastrointestinal tract changes (Figure 1(a)). Ridit analysis showed that the mean *R* values of group A and group B were 0.1650 and 0.1745. Treatment with rhTFF3 by subcutaneous injection significantly decreased the histological injury induced by NEC (Figure 1(b)). Ridit analysis showed that the mean *R* value of group E was 0.3700. In the NEC group, the pathological lesions indicated a severe separation of the submucosa and the lamina propria and tissue necrosis. Most animals had at least some transmural necrosis (Figure 1(c)). Ridit analysis showed that the mean *R* values of group C and group D were 0.7375 and 0.7275. Intestinal damage was significantly less in the rhTFF3 treated rats than in the untreated rats (*P* < 0.01).

### 3.2. rhTFF3 Modulates the Amounts of IL-1*β*, IL-6, and IL-10 in the NEC Model

**Figure 2:**
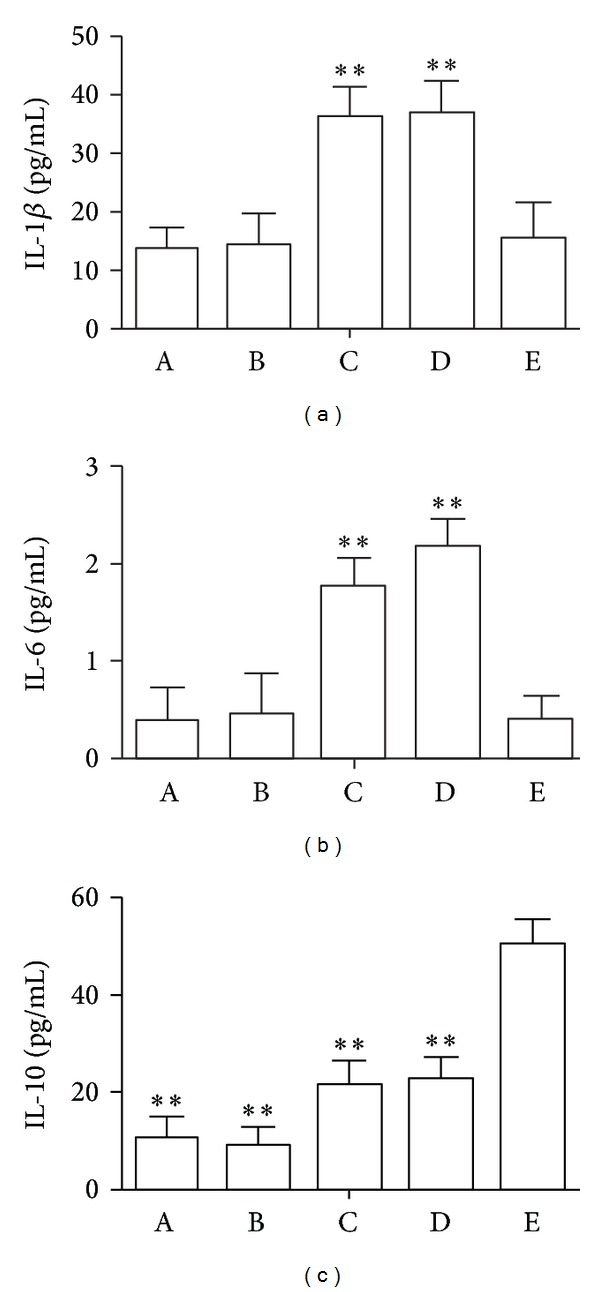
The effect of rhTFF3 on the small intestinal amounts of IL-1*β*, IL-6, and IL-10. (A) Control animal. (B) Control animal treated with rhTFF3. (C) NEC animal. (D) NEC animal treated with 0.9% sodium chloride. (E) NEC animal treated with rhTFF3. *P < 0.05, **P < 0.01 versus the E group.

In the control group, the amounts of IL-1*β* and IL-6 were 13.601 ± 3.782 and 0.364 ± 0.357 pg/mL protein, respectively (*n* = 10). In the NEC group, the amounts of IL-1*β* and IL-6 were significantly higher than in the control group (*P* < 0.01; Figure 2). Treatment with rhTFF3 significantly (*P* < 0.01) decreased the level of IL-1*β* and IL-6 in the NEC model (Figure 2). Treatment with 0.9% sodium chloride by subcutaneous injection did not affect the amounts of IL-1*β* and IL-6 in the NEC model (Figure 2).

In the control group, the amount of IL-10 was 10.600 ± 4.516 pg/mL protein. In the NEC group, the amount of IL-10 was significantly higher than in the control group (*P* < 0.01; Figure 2). Four days after treatment with rhTFF3 by hypodermic injection, the amount of IL-10 was dramatically increased in the NEC model (Figure 2). Treatment with 0.9% sodium chloride by subcutaneous injection did not affect the amount of IL-10 in the NEC model (Figure 2).

### 3.3. rhTFF3 Downregulates the Abundance of NF-*κ*B (p65) in the NEC Model

**Figure 3:**
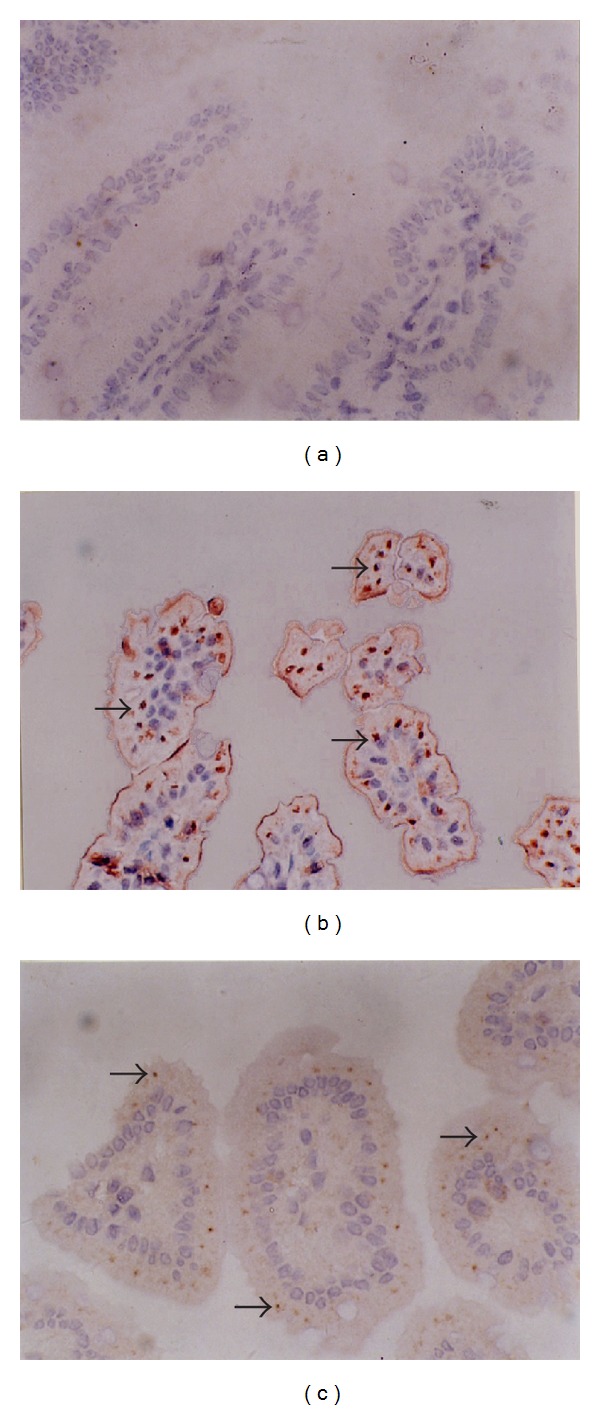
Immunohistochemistry analysis using a polyclonal antibody for NF-*κ*B (Santa Cruz Biotech) in a small intestinal sample from (a) control animal: NF-*κ*B is barely detectable in the epithelium. (b) NEC animal: NF-*κ*B staining is pronounced in the nuclei of inflammatory cells (arrow). (c) NEC animal treated with rhTFF3 by subcutaneous injection for 3 days: NF-*κ*B staining is reduced in the epithelium. All the animals were decapitated and sampled at day 4. (SP, magnification ×400.)

In the normal intestinal tissue, there was no staining or very weak staining for NF-*κ*B (p65) protein (Figure 3(a)). In the NEC model, immunohistochemical staining for NF-*κ*B (p65) was shown as a strong brown color, and it was distributed in the nuclei of the inflammatory cells in the mucosa and villi (Figure 3(b)). Treatment with rhTFF3 by hypodermic injection significantly decreased the immunoreactivity of NF-*κ*B (p65) protein in the NEC model (Figure 3(c)). Treatment with 0.9% sodium chloride by subcutaneous injection did not affect the immunoreactivity of NF-*κ*B (p65) protein in the NEC model (data not shown).

## 4. Discussion

It is widely accepted that NEC is induced by the integrated factors of immaturity, infection, inflammatory reaction, ischemia, hypoxia, improper feeding, and others [14]. All of the factors influence the intestinal mucosa blood supply and local mucosa ischemia and injury, exceeding a necessary threshold for triggering a cascade of inflammatory events. This is sufficient to initiate enteral necrosis, forming NEC. Increasing evidence indicates that, in the complex inflammatory reaction cytokine network, the activation of NF-*κ*B is presumably the key component. As respiratory and enteral inflammation develops, bacterial lipopolysaccharide can induce increased TNF-*α* and IL-1 transcription by activating gland epithelium NF-*κ*B pathways, and the secretion of the cytokines is increased. Because they are also NF-*κ*B reactivators, they trigger leukocyte, macrophage, and lymphocyte NF-*κ*B activation, and NF-*κ*B activation is further augmented via TNF-*α* and IL-1 positive feedback, with a cascade reaction occurring that enables various types of cytokine secretion to be increased by a great quantity and causes aggravated tissue damage [15, 16].

Prodigious expressions of proinflammatory factor IL-8 were found in various types of organs inflammatory reactions. A current study indicates that IL-1*β* mediates IL-8 gene activation by a distinct modality in the IL-1*β*-including intestinal epithelium, the IL-8 promoter gene activation is induced by the NF-*κ*B responsive element [17], and classic NF-*κ*B is a heterodimer composed of RelA (P65) and P50. In a majority of cell types, NF-*κ*B noncovalently binds with its profiling (I*κ*B) and is shielded in the cytoplasm. NF-*κ*B activation is controlled by I*κ*B, which does not require de novo protein synthesis, so rapid and efficient gene regulation can be performed in the enteral epithelium [18]. IL-1*β* causes I*κ*B phosphorylation, switching on I*κ*B rapid degradation and exposing a nuclear localization signal, and the NF-*κ*B (P65) protein is shifted into cell nucleus and bound with the IL-8 promoter gene to induce IL-8 expression.

The upregulation of proinflammatory cytokines, including IL-6, IL-8, and TNF-*α*, can induce and promote inflammation development, and downregulation of the anti-inflammatory cytokine IL-10 leads to insufficient anti-inflammatory action. Both of these play vital roles in NEC formation, and the cytokine expression levels and secretory volume are positively correlated with the degree of NEC inflammation [19, 20]. The demolished balance between pro- and anti-inflammatory cytokines induces and boosts mucosa inflammation, but the eventual intestinal mucosa damage is attributed to phagocyte and granulocyte and their generated inflammation mediators that contribute to macroscopic or microscopic inflammatory bowel disease pathological changes. Anti-inflammatory cytokines (IL-10) play vital roles in the pathogenesis of NEC. IL-10 is generated by monocytes, B cells, helper T lymphocytes, and epithelium [21], and it is able to suppress the synthesis of multiple proinflammatory cytokines and interrupt cell-mediated immune responses [22, 23]. Recent studies indicated that a mouse IL-10 gene knockout via a genetic mutation can induce chronic enterocolitis [24] and effectively hamper IBD formation in the IBD mouse models with persistent IL-10 administration [25].

Previous data indicate that [10], corresponding to normal control enteral tissue, the TNF-*α* and IL-8 content in a NEC model group was remarkably increased, and the levels were significantly decreased in the NEC model group following rhTFF3 treatment. Histopathological examinations indicate that the HE stained sections of the intestinal mucosa of a neonatal NEC model showed that most animals had at least some area with transmural necrosis. All the animals had complete villous necrosis. Treatment with rhTFF3 significantly decreased the histological injury induced by NEC. We propose that hypodermic rhTFF3 injections can relieve enteral inflammatory reactions in neonatal mouse NEC models.

From this study, we found that tissue bowel IL-1*β* and IL-6 amounts were evidently increased, and NF-*κ*B (p65) was activated and underwent nuclear transfer in hypoxia/hypothermia-mediated NEC, indicating that the NF-*κ*B signaling participated in the pathogenesis of neonatal rat NEC and is involved in the function of signal transduction. NF-*κ*B (p65) was weakly positively expressed in the NEC model group following rhTFF3 treatment, and the tissue homogenate IL-1*β* content was decreased, suggesting that rhTFF3 inhibits NF-*κ*B metabolic synthesizing products by inhibiting its expression. The elevations of TNF-*α*, IL-8, IL-1*β*, IL-6, and other cell inflammatory factors were diminished, and anti-inflammatory cytokine IL-10 generation was increased. These show reduced intestinal and colonic tissue inflammatory reactions, and the goal of protecting intestinal mucosa was achieved.

A growing body of evidence suggests that transiently activated NF-*κ*B in noninflammatory states and persistently activated NF-*κ*B during inflammation play different pathophysiological roles in vivo. Transient activation of NF-*κ*B before inflammatory stimulation results in the anti-inflammatory response. Persistent NF-*κ*B activation during the early phase of inflammation amplifies the inflammatory response in vivo. Zhu et al. [26] demonstrated that TFF3-induced NF-*κ*B activation is a transient event. TFF3-induced transient activation of NF-*κ*B is associated with strengthening of the negative regulatory loop of NF-*κ*B [26], which attenuates the inflammatory response via the suppression of NF-*κ*B activity [26, 27]. Recently Loncar et al. [28, 29] showed that the TFF3 expression was significantly reduced in some gastrointestinal cell lines with the overexpression of NF-*κ*B and coexpression of a plasmid constitutively expressing the specific NF-*κ*B inhibitor I*κ*B. Our studies showed that treatment with rhTFF3 reduced NF-*κ*B production. These studies shed light on the role of the cytoprotection factor TFF3 for the inhibition of the proinflammatory mediators NF-*κ*B. More information is needed to determine if the reciprocal regulation of NF-*κ*B by inflammatory cytokines versus protective molecules such as TFF3 is vital to intestinal homeostasis and the appropriate recovery from occasional intestinal injury. In the gastrointestinal (GI) tract, there is accumulating experimental data to suggest that recombinant human TFF3 and recombinant rat TFF3 could serve as valuable pharmacological tools for the prevention and healing of the epithelial barrier and for promoting epithelial restitution after injury in a rat model. Recombinant human TFF3 is the foundation for the commercial production of rhTFF3, and it is necessary to perform further studies to verify the clinical application of rhTFF3 as therapy for NEC patients. A better understanding of the mechanisms underlying these protective effects will be beneficial either in the prevention of NEC or in the development of therapeutic strategies for curing NEC.
